# Gradual onset of the Maunder Minimum revealed by high-precision carbon-14 analyses

**DOI:** 10.1038/s41598-021-84830-5

**Published:** 2021-03-09

**Authors:** Hiroko Miyahara, Fuyuki Tokanai, Toru Moriya, Mirei Takeyama, Hirohisa Sakurai, Kazuho Horiuchi, Hideyuki Hotta

**Affiliations:** 1 Humanities and Sciences/Museum Careers, Musashino Art University, Tokyo, 187-8505 Japan; 2 Faculty of Science, Yamagata University, Yamagata, 990-8560 Japan; 3 Center for Accelerator Mass Spectrometry, Yamagata University, Yamagata, 999-3101 Japan; 4 Graduate School of Science and Technology, Hirosaki University, Hirosaki, Aomori 036-8561 Japan; 5 Department of Physics, Graduate School of Science, Chiba University, 1-33 Yayoi-cho, Inage-ku, Chiba, 263-8522 Japan

**Keywords:** Astronomy and astrophysics, Solar physics

## Abstract

The Sun exhibits centennial-scale activity variations and sometimes encounters grand solar minimum when solar activity becomes extremely weak and sunspots disappear for several decades. Such an extreme weakening of solar activity could cause severe climate, causing massive reductions in crop yields in some regions. During the past decade, the Sun’s activity has tended to decline, raising concerns that the Sun might be heading for the next grand minimum. However, we still have an underdeveloped understanding of solar dynamo mechanisms and hence precise prediction of near-future solar activity is not attained. Here we show that the 11-year solar cycles were significantly lengthened before the onset of the Maunder Minimum (1645–1715 CE) based on unprecedentedly high-precision data of carbon-14 content in tree rings. It implies that flow speed in the convection zone is an essential parameter to determine long-term solar activity variations. We find that a 16 year-long cycle had occurred three solar cycles before the onset of prolonged sunspot disappearance, suggesting a longer-than-expected preparatory period for the grand minimum. As the Sun has shown a tendency of cycle lengthening since Solar Cycle 23 (1996–2008 CE), the behavior of Solar Cycle 25 can be critically important to the later solar activity.

## Introduction

**Figure 1:**
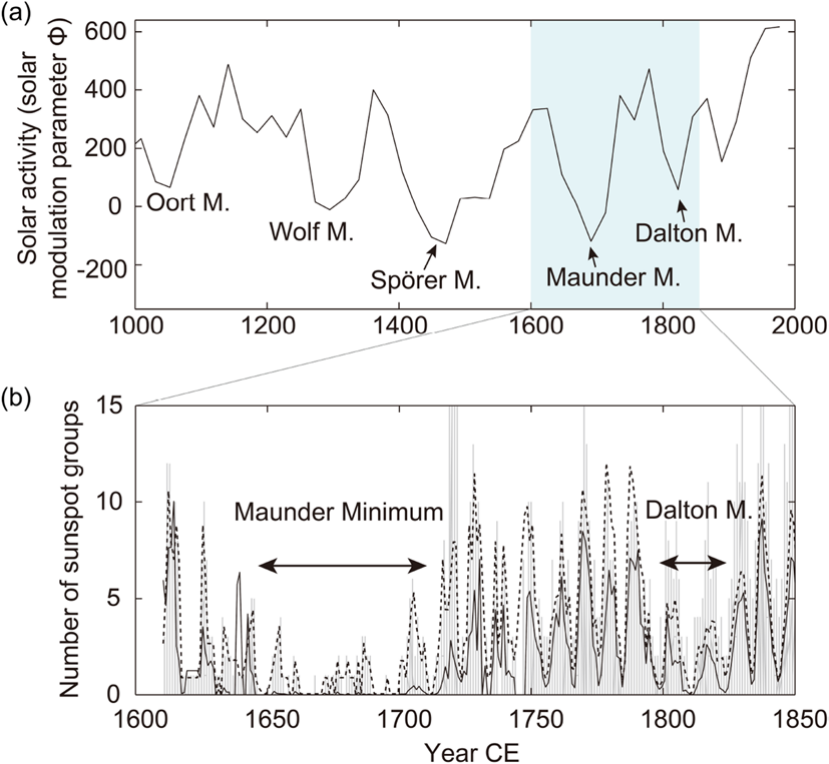
Long-term solar activity during the past millennium. (**a**) Solar activity level reconstructed based on multiple records of cosmic ray induced nuclides^2^. Downward peaks indicate the deep minima in solar activity when the number of sunspots significantly decrease or even disappear from the solar surface. (**b**) Number of yearly mean sunspot groups around the Maunder Minimum (1645–1715 CE) and the Dalton Minimum (1800–1824 CE) obtained by Hoyt and Schatten^4^ (solid line) and by Svalgaard and Schatten^5^ (dashed line), and the number of daily sunspot groups by Vaquero et al.^6^ (gray line) for the same period.

The Sun shows long-term variations with scales ranging from several decades to a few millennia^1, 2^ in addition to the basic decadal-scale cycle, and sometimes brings deep minima in its activity, lasting for several decades or even more than a century^3^. The records of carbon-14 in tree rings or the beryllium-10 in ice cores reveal that the Sun had experienced five such deep minima during the past millennium (Fig. 1a). The galactic cosmic rays shielded by the solar and heliospheric magnetic field produce carbon-14 or beryllium-10; therefore, their production rates reflect the variations of solar activity. The magnitude and duration of solar grand minima are different in each event, and one of the major events is the Maunder Minimum that occurred in the 17th to the early eighteenth century (Fig. 1).

When the Solar Cycle 23 ended end of 2008, nearly 2 years behind the usual rhythm of the 11-year activity cycle, the physical process of the occurrence of grand minima sparked a significant interest. The cycle minimum in 2008 was the deepest in the past 100 years and manifested in many solar-related parameters. Solar wind^7^ and the total solar irradiance^8^ reached the lowest level in recorded history, whereas galactic cosmic rays, shielded by solar and heliospheric magnetic fields, marked the highest level^9^.

As to the possibility of the occurrence of another Maunder-like event, some indications could be, for example, retrieved from directly observing the solar surface. It is well known that the polar magnetic field in the solar cycle minimum highly correlates with the sunspot number in the next solar maximum^10^. The diffusion of the magnetic field of the tilted sunspot pairs and the anisotropic orientation of every scale of the surface magnetic field construct the polar magnetic field^11^. We can predict the polar magnetic field on the basis of the observed sunspot pairs even before the solar minimum by using the surface flux transport model. Several studies have attempted to predict the amplitude of Solar Cycle 25 with this method. Most studies suggest that it would be similar to Cycle 24, whereas some groups suggest a slightly weaker^12, 13^ or stronger^14, 15^ cycle. Note that these predictions accompany uncertainties because of the stochastic nature of the flux emergence. The differences in the predictions, therefore, originate, to some extent, in the treatment of the flux emergence in the models^14, 15^.

Other indications could be derived from a study on past solar cycle variations. It has been suggested that a lengthened solar cycle precedes a decrease in solar activity^16^. Some studies suggest that the lengthening of the 11-year cycle is related to the speed of meridional circulation in the solar convection zone^17, 18^ that could affect the efficiency of the transport or diffusion of the solar magnetic field, although their relationship is unverified.

**Figure 2:**
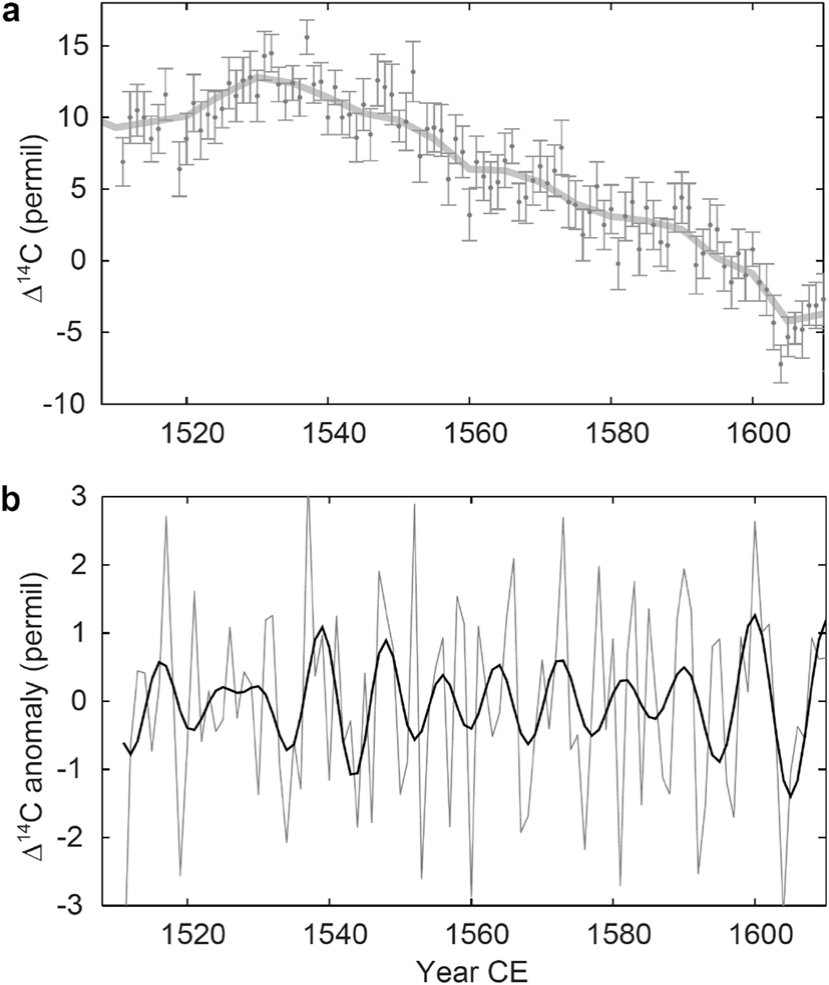
Annual record of carbon-14 before the Maunder Minimum. (**a**) Annual carbon-14 data for 1511–1610 CE obtained by Stuiver, Reimer, and Braziunas^21^ (black dots), and the 5-year-resolution record of IntCal13^22^ (gray, thick curve). (**b**) Band-pass-filtered carbon-14 data in (**a**) with the bandwidth of 8–15 years (thick curve) and 1–15 years (gray line).

Indirect observations of past solar cycles using carbon-14 in tree rings also revealed a similar tendency. Earlier research suggested that the 11-year cycles were lengthened to ~ 14 years during the Maunder Minimum^19^, whereas they were shortened during periods of high solar activity such as the early Medieval Solar Maximum^20^. This coincides with the scenario that the reduction in the speed of meridional circulation could be related to the physical process of the drastic weakening of solar activity. Band-pass-filtered carbon-14 data also show ~ 9-year cycles from 1535 to 1590 CE, which corresponds to when solar activity was relatively high (Fig. 2). Interestingly, later paper pointed out the possibility that the change in cycle length might have started a few cycles before the Maunder or the Spoerer Minima^23^. Although the sunspot groups reconstructed by Hoyt and Schatten^4^ back to 1610 CE had shown a sudden onset of the Maunder Minimum, careful re-examination of historical sunspot records by Vaquero et al.^24^ resolved that the transition into the Maunder Minimum was gradual, with two suppressed activity cycles before the onset. The multiple lengthened cycles suggested by the tree-ring data were consistent with the behavior of sunspot activity cycles, however, the precision of the carbon-14 data was not high enough to determine the accurate timing of the onset of the lengthening or the cycle lengths.

Since then, we improved the precision of carbon-14 data and recently achieved a precision of ~ 0.1%^25^ on the tree ring samples from the Spoerer Minimum. As expected, multiple lengthened cycles were found before the onset of the Spoerer Minimum. However, the records of sunspots are sparse and limited to those by naked-eye observation in the case of the Spoerer Minimum, and thus, a direct comparison with sunspot disappearance cannot be achieved. Investigation of solar cycles before the Maunder Minimum, when sunspot records are available, is indispensable for understanding the process of extreme weakening of solar activity. In this paper, we present solar cycles around the preparatory-period of the Maunder Minimum reconstructed based on unprecedentedly high-precision data of carbon-14, and make a direct comparison with sunspot records.

## Results

**Figure 3:**
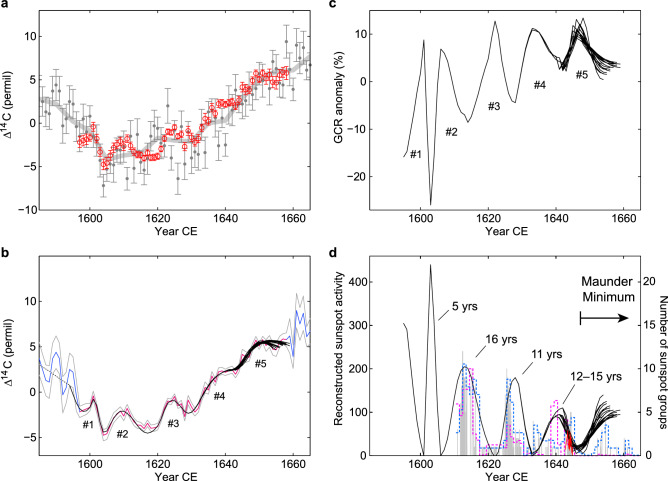
Carbon-14 record and the reconstructed cosmic ray and solar activity cycles around the onset of the Maunder Minimum. (**a**) High-precision annual carbon-14 data obtained in this study (red circles) plotted with the previously obtained annual data by Stuiver et al.^21^ (gray dots) and the 5-year resolution IntCal13 data by Reimer et al.^22^ (gray, thick curve). (**b**) Variation of carbon-14 data used for the reconstruction of solar cycles. Red and gray lines are the high-precision data and their uncertainty ranges. Blue and gray lines are respectively the data and their uncertainty ranges obtained by Stuiver et al.^21^. The black lines show the modeled carbon-14 data that were most consistent with the data (for details on the dashed line, see “Methods” section and Fig. S3). (**c**) Reconstructed cosmic ray variations around the onset of the Maunder Minimum. (**d**) Reconstructed sunspot activity around the studied period (black lines), plotted with the number of yearly mean sunspot groups by Hoyt and Schatten^4^ (pink dashed line) and Svalgaard and Schatten^5^ (blue dashed line). Daily sunspot groups by Vaquero et al.^6^ (gray line) and the monthly mean sunspot groups by Carrasco et al.^28^ for 1642–1645 CE (red line) are also plotted.

Intensely replicated measurements of tree-ring carbon-14 concentrations were conducted using the compact Accelerator Mass Spectrometer installed at Yamagata University in Japan^26, 27^. The measurements achieved a precision of 0.03–0.08% with an average of 0.05%, more than four times better than that usually pursued (for details, see “Methods” section). The obtained data for 1597–1658 CE are, in general, consistent with the previously obtained annual and 5-year resolution data, as shown in Fig. 3a, whereas it reveals distinct decadal-scale cyclic variations that were unclear in the previous dataset.

To reveal the most probable solar and cosmic ray variations for this period, we constructed multiple synthetic cosmic-ray variations with possible parameter ranges, used them as input into the carbon cycle model, and compared the resulted carbon-14 content against the high-precision data (for details, see “Methods” section).

Figure 3c,d show the reconstructed cosmic-ray and solar variations around the onset of the Maunder Minimum, respectively (for details, see “Methods” section). The black curves in Fig. 3b show the range of the modeled carbon-14 variations whose degree of coincidence with the measured data was high. In Fig. 3d, the reconstructed solar cycles are compared with the number of sunspot groups. Note that the height of reconstructed sunspot cycle maximum in Fig. 3d is model-dependent and has some uncertainty for > 270. The variability of reconstructed GCR intensity in Fig. 3c exceeds the range what we have observed during the era of neutron monitors; therefore, we have used the GCR-sunspot relationship extrapolated from the available data (see “Methods” section).

Although the number of sunspot data is limited around this period, we noticed that the evolution of reconstructed solar cycles is consistent with the observational records of sunspot groups in terms of the relative amplitudes and the timing of the cycle minima, especially for the first two sunspot cycles since telescopic observations began (Fig. 3d). Recently, there have also been several researches reconstructing sunspot butterfly diagram for this period^29–33^. According to those reconstructions, high-latitude sunspots, which can be the sign of the arrival of new solar cycle, had started to appear around the end of 1621 CE and 1633 CE. The sunspot cycle minima reconstructed based on carbon-14 are in 1622 CE and 1633 CE, which are consistent with the reconstructed butterfly diagram. Note that some of the high-latitude sunspots may start to appear a few months earlier than the actual onset of solar cycle i.e. sunspot minimum of 13-month moving averages, and that the timing of cycle minima suggested by the butterfly diagram may have uncertainties less than a few months. For the pre-telescopic era, historical aurorae records can be used to examine the validity of solar cycles reconstructed by carbon-14. Note, however, that peaks in auroral activity may lag sunspot cycle maxima by a few years^34^. The minima of reconstructed solar cycles shown in Fig. 3d are in 1601 and 1606 CE, and we find that both correspond to the period the number of reported aurorae is small (see Fig. 8 of Váquez et al.^35^). The agreement between the sunspot/auroral data and the reconstructed solar cycles based on carbon-14 confirms that carbon-14 assists in tracing past solar cycles, although the variations are significantly attenuated in the carbon cycle and, thus, there is need for high-precision measurements.

Carbon-14 record with improved precision achieved in this study allowed us to discuss the length of each solar cycle. We found that the solar cycle that started around 1601 CE lasted about 5 years, much shorter than the mean length of solar cycles. However, the subsequent cycle shows a distinct lengthening, suggesting that this cycle was lengthened to about 16 years, approximately 5 years longer than the average. Note that the mean cycle length since 1755 CE is 11.02 ± 1.2 years^36^. The subsequent cycle then seems to be about 11 years. The length of the cycle just before the Maunder Minimum again seems to be lengthened to be about 12–15 years. We found that the data of the following cycle significantly constrain determining the cycle length (see “Methods” section). Extending the high-precision data, therefore, is needed to narrow the estimation range for this cycle.

## Discussion

An important finding of this study is that the lengthening of solar cycle started three cycles before the onset of the Maunder Minimum. In the framework of the flux transport dynamo model, which is known to reproduce several features of solar cycle, solar activity level is determined by either or both of two factors: dynamo excitation by the randomly determined tilt of sunspot pairs^37^ and the change in the meridional circulation in the solar convection zone^38^. On the one hand, the flow speed of meridional circulation determines the cycle lengths^17^, although its structure is still controversial^39^. Under the condition the time-scale of turbulent diffusion of the magnetic field in the convection zone is relatively short, slow meridional circulation could cause a substantial loss of the magnetic field. One possible interpretation of the multiple lengthened cycles before the Maunder Minimum is that the speed of meridional circulation was significantly slowed down to contribute to the reduction of the magnetic field that emerges on the solar surface as sunspots. The reconstructed variation of cosmic rays in Fig. 3c certainly suggests that the intensity of the solar surface magnetic field at the end of the 16 year-long cycle became significantly weakened compared with the previous solar cycle minimum. The absolute levels of sunspot activity over the subsequent two cycles needs to be determined through the ongoing efforts to discover additional historical records and to improve the methodology of reconstruction^31^; however, the sunspot reconstructions during the recent decade have indicated a tendency of gradual reduction in the cycle amplitudes toward the Maunder Minimum^5, 6, 24, 31^ and are consistent with our results.

The long preparatory period observed at the Maunder Minimum is consistent with what was suggested for the Spoerer Minimum. It is also noticeable that one of the preceding cycles of the Spoerer Minimum was ~ 16 years^25^, although this estimation is based on band-pass filtering and could have large uncertainty. On the other hand, only one cycle was lengthened before the onset of the Dalton Minimum, which was 13.6 years^16^. The Dalton Minimum is different from the Maunder and the Spoerer minima regarding its duration and depth. We hypothesize that the lengthening of plural neighboring solar cycles, among which at least one cycle is several years longer than 11 years, could be a prerequisite for long-lasting sunspot disappearance.

While the length of Solar Cycle 23 was 12.7 years, ~ 2 years longer than usual, the Solar Cycle 24 did not show a significant lengthening. Therefore, current declining tendency in solar activity is less likely to immediately result in a long-lasting sunspot disappearance. We conclude, however, that the behavior of Solar Cycle 25 would be critically important to the later solar activity and that there remains the possibility that sunspots may disappear for decades in the case Solar Cycle 25 is substantially lengthened. Careful examinations of both the solar surface and the interior are needed throughout the Solar Cycle 25.

Our current understanding of solar dynamos will predict the change in meridional circulation only when a large-scale magnetic field is developed to disrupt the flow by the Lorentz force, angular momentum transport, or by the changing pressure balance due to the sunspot emergence^40^. However, the sunspot peak of the 16 year-long cycle is not outstandingly high; instead, the preceding cycle shows a significant enhancement in the magnetic activity. The lagged reduction in the meridional circulation, therefore, is a theoretical challenge to be solved in the future.

### Data set

The data set that supports the findings of this study is listed in Table S1.

## Methods

### High-precision measurement of carbon-14

We used the compact Accelerator Mass Spectrometer (AMS) installed at the Yamagata University^26, 27^ for the measurement of carbon-14 content in tree rings. We used two cedar tree samples for this study. One is a 382 year-old cedar tree (Cryptomeria japonica) obtained at the Murou temple at the Nara prefecture in Japan (34° 32′ N, 136° 02′ E). This sample covers 1617–1998 CE and was previously used to reconstruct the solar cycles during the Maunder Minimum^19^. The other is a 439 year-old cedar tree obtained at the Ise grand shrine in the Mie prefecture (34° 27′ N, 136° 44′ E). This tree covers 1501–1959 CE^41^. The trees were subdivided into blocks, and each of the annual tree rings was separated to produce graphite as the target material of AMS following the procedures presented by Moriya etal.^25, 27^.

To achieve a high precision of < 0.08%, we conducted intensely replicated measurements for a relatively short period (i.e. from 1597 to 1658 CE). We introduced into AMS three to four cathodes filled with graphite produced from each of the annual samples and ran the measurements for each target wheel for 14 cycles (300 s for each cycle). Then, if the conditions of cathodes and the AMS allowed it, we repeated the measurements twice (10–14 cycles). We replicated such measurements several times until the uncertainty of Δ14C becomes smaller than 0.08%. Two blanks prepared from JAEA-C1 and approximately nine standard samples (JAEA-C6 and NIST standard) were installed in one target wheel to accurately calculate Δ14C and monitor the systematic errors of the measurements.

To reduce the systematic errors attributed to the instability of AMS and minimize the error bars of the carbon-14 data, we treated each of the 14 cycles as completely different measurements. Δ14C of the samples were obtained for each cycle by normalizing the activity by those of the standard samples at the same cycle. Furthermore, we conducted delta 13C correction for every 0.5 s (600 steps for 300 s) to calculate Δ14C. We then obtained the weighted means of Δ14C for each of the annual samples. Table S1 lists the high-precision data obtained from eight years of measurements from 2013 to 2020.

Figure S1a displays the weighted mean of Δ14C for the Murou and Ise samples (black and blue circles), respectively. The red circles in Fig. S1b show the weighted mean for all of the measurements. The gray circles in Fig. S1a are the previously obtained biennial data using the Murou and Yaku cedar samples by Miyahara et al.^19^ and Miyahara et al.^42^, and the gray dots in Fig. S1b are the annual data previously obtained by Stuiver et al.^21^. The newly obtained high-resolution data are consistent with the IntCal13 data^22^ and the annual data by Stuiver et al.^21^ within the measurement uncertainties. Note that the long-term trend in the carbon-14 content is attributed to the long-term variation of solar magnetic activity and the resultant increase of the carbon-14 production rate. Because of the cumulation of carbon-14 in the atmosphere, it shows an upward trend while the solar magnetic activity is in a relatively weak condition.

### Methodology of reconstructing solar cycles based on the high-precision data

On the basis of the high-precision data as obtained above, we reconstructed the solar cycles around the onset of the Maunder Minimum. The procedure is as follows: (1) construct model curves for sunspot cycle, (2) construct correspondent cosmic-ray variations, (3) solve the three-box carbon cycle model to derive the resultant atmospheric carbon-14 variations, and (4) compare them with the high-precision carbon-14 data.

A straightforward way to reveal the decadal-scale variation of cosmic rays from the carbon-14 data is to take the differentials of the annual data, solve the carbon cycle model with reverse time according to the carbon-14 budget equation [Eq. (1) in Ref.^43^], and derive the production rate of carbon-14; however, any wiggles in the carbon-14 data, including the ones associated with the measurement uncertainty, would be largely reflected to the reconstructed variation of cosmic rays because the attenuation rate of carbon-14 variation in the atmosphere is especially large for short-term variations (see Fig. 5 of Ref.^43^). We, therefore, decided to solve the carbon cycle model forward with multiple scenarios of cosmic ray variations. We input the variation of carbon-14 production rate equivalent to the synthetic cosmic ray variations into the carbon cycle model, and compared the resultant atmospheric carbon-14 variation with the high-precision data. In this way, we determined the profile of cosmic ray variations that could well explain the observed carbon-14 and then estimated the most probable variations for solar cycles.

First, we constructed a model curve for the sunspot cycle. Here, we used the sunspot data back to 1712 CE^5^. By normalizing the sunspot cycles both by the peak numbers and cycle lengths, we averaged the 27 sunspot cycles since 1712 CE to construct a typical curve for the sunspot cycle. Then, we constructed synthetic curves for the sunspot cycle. We treated the following four parameters as variables: (1) sunspot number at the cycle maximum, (2) sunspot number at the cycle minimum, (3) cycle length, and (4) the length of the declining phase. We constructed sunspot cycles with these parameters starting from the sunspot maximum (cosmic-ray minimum) and obtained the corresponding cosmic-ray variations, as described below.

Second, we constructed a model curve for the cosmic-ray variation. Figure S2a indicates the sunspot numbers since 1953^5^, as well as the neutron monitor data obtained at Oulu^44^ and Climax^45^. Both of the data were normalized, and the Climax data were scaled to the Oulu neutron data (Fig. S2b) to average the two series. The combined neutron monitor data (Fig. S2c) were then compared with the sunspot data. The solar magnetic polarity reverses at every maximum of sunspot decadal-scale cycle, and this polarity influences the trajectory of galactic cosmic rays in the heliosphere. The time profile of the cosmic ray variation, therefore, is dependent on the polarity of the Sun. Figure S2d indicates the relationship between cosmic rays and the sunspot numbers for positive (red circles) and negative (blue hexagrams) polarities. On the basis of the relationship, we constructed a simple model (red and blue lines) to construct the curves of cosmic-ray cycles from the sunspot activity cycles. For the positive polarity, we used the second-order approximation of the data. For the negative polarity, we used the first-order approximation for sunspot numbers < 30, whereas we applied the second-order approximation for ≧ 30. For the negative polarity, the annual data of beryllium-10 suggested that the cosmic-ray flux could be enhanced by up to 30–40% at a very weak condition of solar and heliospheric magnetic activity, associated with a change in the large-scale structure of the heliospheric current sheet^46^. We, therefore, extrapolated the curves for sunspot numbers < 0 to consider the cases of cosmic-ray flux exceeding the level of 2008–2009 CE either by the further weakened solar magnetic field or by the change in the heliospheric structure. For the sunspot number > 270, we used the extrapolation of the red line for both positive and negative polarities. When solar activity is high, transient events such as solar coronal mass ejections significantly influence the reduction of cosmic rays on the Earth. The influence of solar polarity, therefore, becomes small. On the basis of these two curves, we constructed time profiles of cosmic-ray variations which were used as input to the carbon cycle model.

Finally, we solved the carbon cycle model with multiple possible curves of cosmic-ray variations as input. We used the three-box carbon cycle model with carbon-14 exchange rates presented by Roth and Joos^43^. As a setup, we constructed a long-term cosmic ray curve by taking 7-point moving averages of the 5-year resolution data of IntCal13^22^(gray, thick line in Fig. S3a) and by inversely solving the carbon cycle model. Figure S3b shows the obtained long-term variation of cosmic rays. To this variation, we connected the synthetic curves of cosmic ray cycles starting around 1591–1596 CE to run the carbon cycle model. Previous study has suggested that carbon-14 peak around 1671–1673 corresponds to solar cycle minimum of negative polarity^46^. It allows to estimate that carbon-14 peaks around 1601, 1623, and 1649 CE as are seen in Fig. S3a correspond to solar cycle minima of negative polarity, while the peaks around 1609 and 1636 correspond to positive polarity. Based on this estimation, we constructed synthetic cosmic-ray curves starting around 1591–1596 CE with negative polarity.

In this study, we assumed that the possible ranges for the above four parameters are (1) 40–440, (2) ≦ 40, (3) ≧ 7 years, and (4) ≧ 3 years. We also assumed that the minimal length of the ascending phase of the solar cycle is 2 years. The limitations on the ascending and the declining phase of the cycles were determined based on the evolution of the sunspot cycles since 1712 CE (Fig. S4). We calculated atmospheric carbon-14 content with the three-box carbon cycle model by inputting the scenarios with the above parameters in steps: (1) 5, (2) 5, (3) 1 year, and (4) 1 year. Then, to examine the degree of coincidence between the modeled carbon-14 and the measured ones (blue and red lines in Fig. S3a), we calculated the chi-square values X^2^ = ∑ (Δ^14^C_modeled_ − Δ^14^C_measured_)^2^/σ^2^ for each calculated cycle, where σ is the uncertainty of the measured carbon-14.

### Results of the carbon cycle modeling

In this study, we made comparisons between the modeled and measured data for each cosmic-ray cycle, starting from sunspot maximum to the next maximum. We first conducted the calculation of chi-square values for the cycle starting around 1591–1596 CE (Cycle #1). For this calculation, we used Stuiver et al.’s^21^ previously obtained annual carbon-14 data and their uncertainties (thick and thin blue lines in Fig. S3a, respectively) before 1597 CE besides our high-precision data from 1597 CE.

Figure S5a shows the chi-square values for Cycle #1. The dots indicate the lowest chi-square values for each case of the starting year (Yst) and cycle length. On the basis of these results, we determined the most probable length of Cycle #1. We found that the chi-square value is the smallest when the length of Cycle #1 is 8 years and Yst is 1595.

For Cycle #2, we calculated the chi-square values for the cases Yst is 1591–1596 (Cycle #1 is 12 to 7 years, respectively) which is shown in Fig. S5b. The consistency between the results confirms that Yst within this range does not significantly affect the reconstructions. Here we then focus on the results calculated with Yst = 1595.

The chi-square values for Cycle #2 illustrates that the modeled curves correspond well with the measured data for period ≦ 13 years, and that the calculation on this cycle alone does not strongly constrain the length of Cycle #2. We found that the calculation of the subsequent cycle (Cycle #3) gives a stronger constraint on Cycle #2 (Fig. S5c). Most of the cases are rejected by a 95% confidence level except the case calculated with Cycle #2 as 10 years. Similarly, the calculation of Cycle #4 gives a strong constraint on the length of Cycle #3. In the case of Cycle #4, the lowest chi-square values are achieved in the case Cycle #3 as 15 years (Fig. S5d). The constraint on the cycle length is also given by the subsequent cycle because, even though the modeled curve fit the measured curve within the data uncertainties in one cycle, in some cases, none of the possible curves with the given parameter ranges could reproduce the subsequent cycle well.

Figure S5e indicates the results for Cycle #5, under the condition that the cycle length of Cycles #1, #2, and #3 are 8, 10, and 15 years, respectively. The relatively low values achieved in the case of Cycle #4 is 12, 13, and 14 years. Although insignificant, troughs are seen in the results for Cycle #5 around 10–18 years. Figure S5f–h show the results of Cycle #6 obtained for the cases of Cycle #4 as 12–14 years, and Cycle #5 as 10–18 years. We found that, in the case of Cycle #5, no strong constraints are given from the calculation of Cycle #6. On the basis of the chi-square values for Cycles #5 and #6, we regard the possible ranges for Cycle #5 as 14–18 years (in the case Cycle #4 = 12 years), 13–18 years (in the case Cycle #4 = 13 years), and 12–17 years (in the case Cycle #4 = 14 years).

The uncertainty in determining the cycle length is relatively large for Cycle #4 compared with Cycles #1 to #3, and it becomes even larger in the case of Cycle #5. We assume that it is because of the suppression of the decadal-scale variation around the time as well as the relatively large errors of our data toward the end of the series. Furthermore, for Cycle #5, a strong constraint could not be given by the subsequent cycle because high-precision data are not available.

Figure 3c,d show the reconstructed cosmic-ray and sunspot variations based on the obtained results. As mentioned above, the determined cycle lengths, based on Fig. S5, are the ones counted from the sunspot maximum to the next maximum; therefore, we manually counted the lengths of the solar cycles from the sunspot minimum to minimum. The estimated cycle lengths for the solar cycles starting around 1601 CE are 5, 16, 11, and 12–15 years, as summarized in Fig. 3d.

## Supplementary Information

Supplementary Information
