# Supplementary material for: Gradual onset of the Maunder Minimum revealed by high-precision carbon-14 analyses
Source: Sci Rep. 2021 Mar 9;11:5482. doi: 10.1038/s41598-021-84830-5 (PMC7943760; doi:10.1038/s41598-021-84830-5)
Supplement: Supplementary file 1 — Supplementary Information [file 41598_2021_84830_MOESM1_ESM.pdf]

Supplementary Information

Gradual onset of the Maunder Minimum revealed by high-precision carbon-14 analyses

Hiroko Miyahara*, Fuyuki Tokanai, Toru Moriya, Mirei Takeyama, Hirohisa Sakurai, Kazuho Horiuchi, Hideyuki Hotta

Table S1: List of carbon-14 data obtained during 2013 to 2020 using the AMS at Yamagata University

Year CE	$\Delta^{14}C$ (permil)	Errors (permil)	Number of measurements
1597	-2.18	0.62	4
1598	-2.00	0.62	4
1599	-1.85	0.60	4
1600	-1.66	0.41	6
1601	-0.41	0.51	6
1602	-1.78	0.49	6
1603	-3.32	0.49	6
1604	-4.75	0.61	3
1605	-4.59	0.63	4
1606	-3.70	0.56	3
1607	-2.83	0.56	3
1608	-2.45	0.49	5
1609	-2.08	0.46	6
1610	-2.64	0.51	5
1611	-1.79	0.44	5
1612	-2.60	0.43	5
1613	-3.27	0.42	6
1614	-3.62	0.38	8
1615	-3.66	0.33	8
1616	-4.02	0.32	9
1617	-3.43	0.35	8
1618	-3.98	0.35	8
1619	-3.94	0.38	8
1620	-3.81	0.36	8
1621	-2.96	0.33	10
1622	-1.78	0.32	10
1623	-0.98	0.31	9
1624	-1.07	0.31	11
1625	-0.47	0.29	11
1626	-1.50	0.30	11

1627	-1.27	0.37	8
1628	-2.70	0.47	5
1629	-0.88	0.52	5
1630	-1.63	0.47	5
1631	-2.20	0.43	5
1632	-1.77	0.46	5
1633	-0.54	0.43	5
1634	0.52	0.61	3
1635	0.23	0.45	5
1636	1.78	0.49	5
1637	1.10	0.40	7
1638	2.24	0.44	7
1639	2.34	0.38	7
1640	2.18	0.35	9
1641	2.35	0.37	8
1642	2.47	0.40	7
1643	1.97	0.39	7
1644	2.65	0.46	4
1645	4.16	0.56	3
1646	3.87	0.60	3
1647	3.83	0.43	5
1648	4.92	0.42	5
1649	5.73	0.40	5
1650	5.07	0.35	7
1651	5.78	0.36	7
1652	5.13	0.64	3
1653	5.59	0.65	3
1654	4.73	0.65	3
1655	4.66	0.73	3
1656	5.54	0.62	3
1657	5.86	0.58	4
1658	5.78	0.75	3

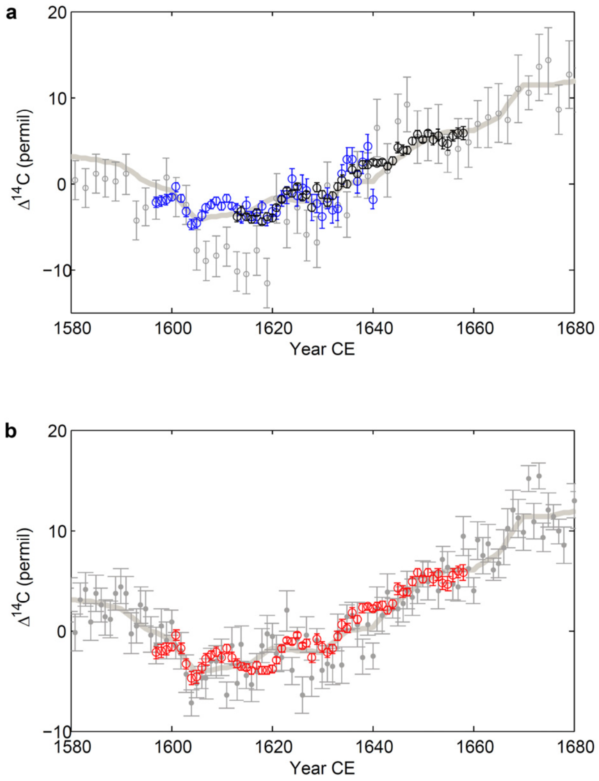

Figure S1: Carbon-14 content in tree rings around the onset of the Maunder Minimum. (a) High-precision annual carbon-14 data obtained using the Murou cedar tree (black circles) and Ise cedar tree (blue circles), plotted with the previously obtained biennial data (gray circles) using the Murou cedar tree[19] (after 1631 CE) and Yaku cedar tree[42] (before 1629 CE). The gray, thick curve indicates the 5-year resolution carbon-14 records of IntCal13[22]. (b) Compiled carbon-14 data for 1597–1658 CE (red circles) plotted with the previously obtained annual carbon-14 data by Stuiver et al. (1998) (gray dots) and IntCal13 records[22] (gray, thick line).

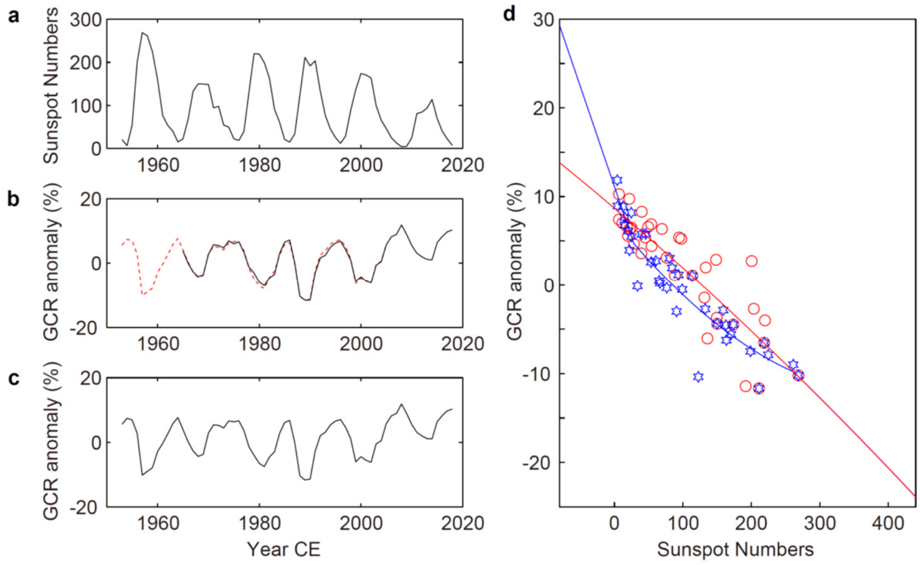

Figure S2: Variations of sunspot numbers and the galactic cosmic rays. (a) Sunspot numbers since 1953 CE[5]. (b) Variation of galactic cosmic rays as measured by the Oulu neutron monitor[44] (black curve) and the Climax neutron monitor[45] (red dashed line). Note that the Climax data are scaled to the Oulu data. (c) The combined curve of the galactic cosmic rays. (d) Comparison between the cosmic ray anomaly and the sunspot numbers since 1953 CE for the two phases: the polarity of the solar dipole magnetic field is positive (red circles) and negative (blue hexagrams), respectively. The red and blue curves are the model curves used in this study.

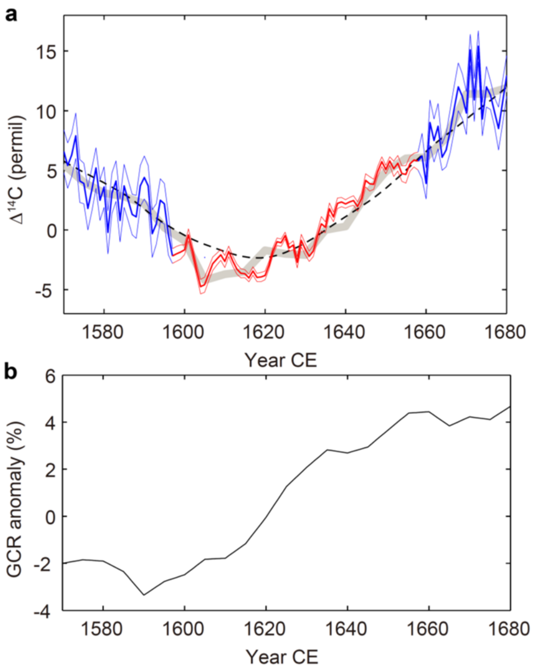

Figure S3: Variation of carbon-14 data used in this study. (a) Thick and thin red lines are the high-precision data and their uncertainties obtained in this study. The data by Stuiver et al. (1998) and their uncertainties, as shown by thick and thin blue lines, respectively, were also partially used for the period that our data are unavailable. The gray, thick line indicates the 5-year resolution carbon-14 data by Reimer et al. (2013). (b) The long-term trend of cosmic rays calculated by the carbon cycle model using the 7-point moving averages of the gray, thick line in (a). The long-term atmospheric carbon-14 variation resulting from this curve is shown in panel (a) by the black dashed line.

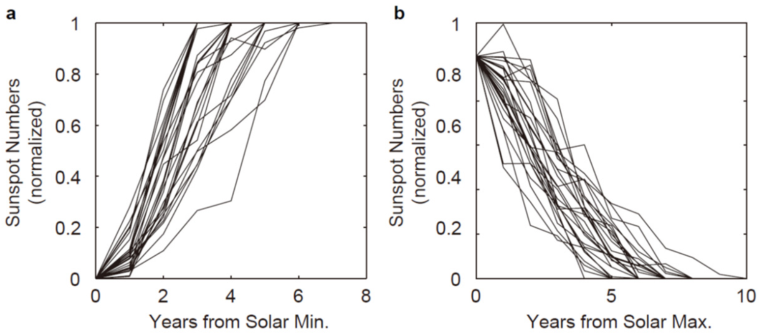

Figure S4: The evolution of sunspot cycles analyzed based on the sunspot data since 1712 CE[5]. (a) The number of years needed to reach to the sunspot cycle maximum. (b) The number of years needed to reach to the sunspot cycle minimum counting from the activity maximum. Note that the sunspot peaks are normalized to 1.

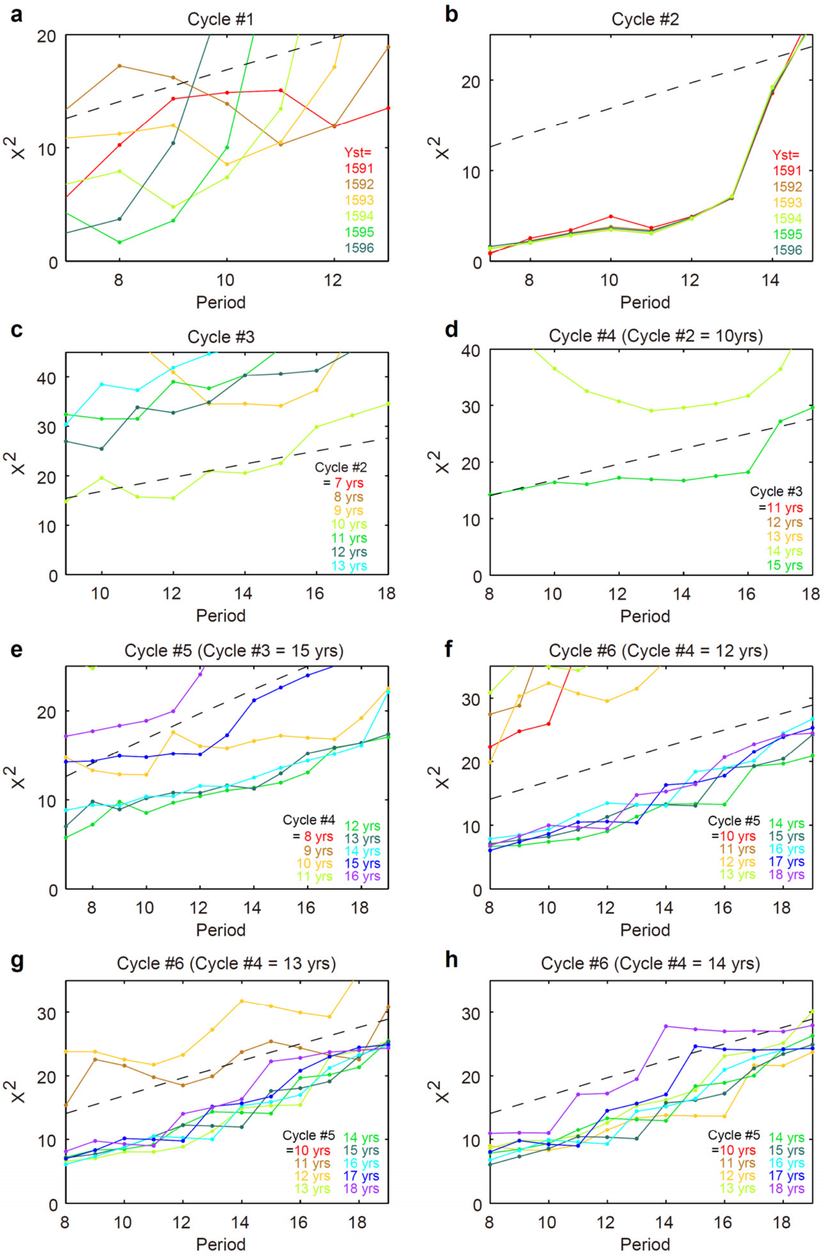

Figure S5: The chi-square values for Cycles #1 to #6 obtained from the comparison between the modeled and measured carbon-14. Note that Cycle #1 is assumed to be negative polarity. The areas above the black dashed lines are rejected with a 95% confidence level. Note that the labeled but not shown lines are above the limit.
